# Physical Ageing of Amorphous Poly(lactic acid)-Indapamide System Studied by Differential Scanning Calorimetry

**DOI:** 10.3390/pharmaceutics15092341

**Published:** 2023-09-19

**Authors:** Marcin Skotnicki, Agata Drogoń, Janina Lulek, Marek Pyda

**Affiliations:** 1 Chair and Department of Pharmaceutical Technology, Poznan University of Medical Sciences, 60-780 Poznan, Poland; marcskot@ump.edu.pl; 2 Department of Chemistry, Rzeszow University of Technology, 35-959 Rzeszow, Poland

**Keywords:** physical ageing, enthalpy relaxation, indapamide, poly(lactic acid), differential scanning calorimetry, Kohlrausch–Williams–Watts equation, fragility

## Abstract

The process of isothermal and non-isothermal physical ageing of amorphous polylactide (PLA) with the active pharmaceutical ingredient, indapamide (IND), was investigated. A PLA–IND system with a 50/50 weight ratio was obtained and characterized using differential scanning calorimetry (DSC). In the 50/50 (*w*/*w*) mixture, two glass transitions were observed: the first at 64.1 ± 0.3 °C corresponding to the glass transition temperature (*T*_g_) of PLA, and the second at 102.6 ± 1.1 °C corresponding to the *T*_g_ of IND, indicating a lack of molecular mixing between the two ingredients. The PLA–IND system was subjected to the isothermal physical ageing process at different ageing temperatures (*T*_a_) for 2 h. It was observed that the highest effect of physical ageing (enthalpy relaxation change) on IND in the PLA–IND system occurred at *T*_a_ = 85 °C. Furthermore, the system was annealed for various ageing times at 85 °C. The relaxation enthalpies were estimated for each experiment and fitted to the Kohlrausch–Williams–Watts (KWW) equation. The KWW equation allowed for the estimation of the relaxation time and the parameter describing the distribution of relaxation times of the isothermal physical ageing process of IND in the PLA–IND system. The physical ageing of the PLA–IND mixture (50/50) was also discussed in the context of heat capacity. Moreover, the activation energy and fragility parameters were determined for the PLA–IND (50/50) system.

## 1. Introduction

Active pharmaceutical ingredients (APIs) may exist in a crystalline or amorphous form. The crystalline state is characterised by a regularly ordered lattice structure. In practical terms, the structures of these systems are generally thermodynamically stable and are relatively simple to study using techniques such as differential scanning calorimetry (DSC) or X-ray diffraction methods. On the other hand, there is no long-range order to amorphous forms and their “structures” are not easy to characterise by standard X-ray diffraction methods. A valuable technique for studying amorphous pharmaceuticals is DSC [1,2,3]. Amorphous drugs usually dissolve more readily and are more bioavailable than their crystalline counterparts [2]; however, amorphous APIs may recrystallise during the shelf-life of the formulation [4]. A common approach to stabilise amorphous drugs is polymeric amorphous solid dispersions (PASDs) [5]. In PASDs, the improved stability of an amorphous API is achieved by entrapping the drug in a high-energy glassy state between the polymer chains [6,7]. Although the excipients are often considered inert, it is known that they can interact with APIs, changing their stability, absorption and bioavailability [8,9]. Therefore, APIs must be investigated during preformulation studies at the early phase of the drug development process, in order to provide the necessary information to develop a stable formulation with increased bioavailability [10].

The amorphous forms of API may be desirable due to their improved apparent solubility and, as a consequence, their bioavailability in comparison with its crystalline counterparts. However, in contrast with crystals, glasses are not thermodynamically stable [11,12,13,14,15]. Thus, the stability of amorphous APIs is a primary issue associated with their use in the formulation. The amorphous forms of APIs, during storage below or above the glass transition temperature *T*_g_, may revert to the crystalline form [6,11,16], losing their superior properties. Below the glass transition temperature and above a Kauzman temperature, glasses undergo a physical ageing process, i.e., structural relaxation towards thermodynamic equilibrium as a function of time and temperature [17]. In contrast with chemical or biological ageing, physical ageing is a reversible phenomenon involving the ordering of the amorphous phase, during which no breaking or forming of chemical bonding occurs. More significant structuring may cause, among other things, the deterioration of solubility [18], diffusivity and permeability [19], a decrease in physical stability [20], and a change of mechanical properties [21,22]. For instance, a decrease in solubility has been observed for physically aged cinnarizine-Soluplus solid dispersions [18]. Annealing may also positively affect amorphous APIs, for instance, it can increase chemical stability [23].

In this work, a polymer–drug system was obtained using amorphous poly(lactic acid) and an amorphous API—indapamide.

Poly(lactide) (PLA) belongs to the group of aliphatic polyesters. Depending on D-, L-isomers content, it can exist in a semi-crystalline or an amorphous state exhibiting different physicochemical properties [24,25,26]. It is widely used in the food, pharmaceutical and medical industries. In pharmaceutical applications, PLA is used as a drug carrier matrix in drug delivery systems as well as the bulk component of medical devices due to its biocompatibility and bioresorbability [27,28,29]. PLA is widely used in formulations in order to modify the dissolution profile of an API or to improve its stability [30,31]. The drug can be released from the polymer matrix in a controlled and prolonged manner. For example, Leroueil-Le Verger et al. successfully used solid dispersion with polylactide in an oral controlled release system for isradipine [30].

Indapamide (IND) is a thiazide-like diuretic drug used to treat hypertension [32]. IND is practically insoluble in water [33] and belongs to the biopharmaceutics classification system class II (low solubility, high permeability). Indapamide available on the market is formulated in a crystalline form, e.g., [34], however, it can also be obtained in an amorphous form [33,35,36].

The ability of a material to transform into its amorphous state is called glass-forming ability (GFA) [37]. APIs are categorised into three classes, I, II and III, based on their GFA [37,38]. The GFA of indapamide belongs to class III, where the material, after melting the crystalline form, does not recrystallise during the DSC cooling/heating cycle, and the sample remains amorphous.

**Figure 1:**
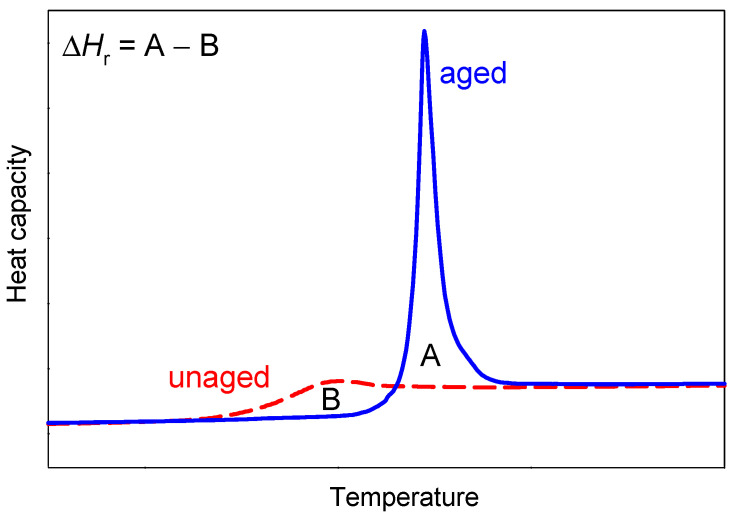
Scheme for determination of enthalpy relaxation from DSC plot of heat capacity vs. temperature.

As previously mentioned, amorphous materials, unlike crystals, are in a thermodynamic, non-equilibrium state, and, therefore, such materials undergo the physical ageing process. The process is a physical phenomenon related only to the amorphous solid phase. At a temperature lower than the glass transition temperature, the amorphous materials gradually evolve toward the thermodynamic equilibrium of the glassy state [17]. The aged amorphous material tends to be in a more equilibrium state, with less energy [39,40,41]. The physical ageing process can be studied, among other things, using differential scanning calorimetry [36,42]. On the scan obtained from the standard DSC measurement of an unaged amorphous material, a glass transition with a change in specific heat is observed in the heat flow or heat capacity versus temperature plot (Figure 1). When the amorphous sample is aged isothermally at a temperature lower than the glass transition temperature (called the ageing temperature) and is reheated through the glass transition region, an endothermic peak in the DSC curve is observed in addition to the change of heat capacity (Figure 1).

The physical ageing process can be described by measuring the enthalpy relaxation or enthalpy recovery, Δ*h*_r_, which manifests an order in the amorphous structure and molecular mobility [17,43,44]. Enthalpy relaxation depends on the sample’s thermal history and is the additional energy burden required to reverse the ageing process—to destroy/reverse the ordering resulting from the evolution of the glass towards equilibrium. It can be calculated from the area difference between the heat flow or heat capacity curves of aged (A) and unaged (B) samples (Δ*H*_r_ = A − B) (see Figure 1). The relaxation kinetic of the non-equilibrium glassy state can be determined using various models [43]. One of these models is the Kohlrausch–Williams–Watts model (KWW) [45,46].

One of the parameters describing the molecular mobility of the amorphous material is the fragility parameter (*m*). The fragility parameter has been used in the pharmaceutical industry as it can evaluate the tendency of amorphous API towards recrystallisation [14]. In accordance with the classification introduced by Angell, glasses can be referred to as “strong” or “fragile”, depending on the value of *m* [47]. “Strong” glasses are characterised by a low value of *m*, while “fragile” materials have a higher *m* value. Glasses classified as strong are expected to be more physically stable than fragile ones [48].

Heat capacity is an important quantity obtained by DSC for the characterisation of the thermal properties of amorphous systems. Quantitative thermal analysis of non-equilibrium processes and states needs reference baselines of solid (vibrational heat capacity) and liquid heat capacity [49,50,51]. These can be determined for complex systems using a linear combination of the vibrational heat capacity of each ingredient with their molar or weight fractions [49].

The aim of this work was to investigate the amorphous system of API (indapamide) with polymer (polylactide) in the 50:50 weight ratio. The system was not molecularly mixed—two glass transitions were detected. PLA–IND was comprehensively characterized by the differential scanning calorimetry. The process of isothermal physical ageing of IND in the binary PLA–IND system is shown for the different ageing temperatures and times. The kinetics of ageing of a two-component system was analysed by the Kohlrausch–Williams–Watts model. The data for aged and unaged samples of PLA–IND are presented in the frame of reference solid and liquid heat capacity. Furthermore, the activation energies of the structural relaxation at glass transition temperature and fragility parameters were determined for the system.

## 2. Materials and Methods

### 2.1. Materials

**Figure 2:**
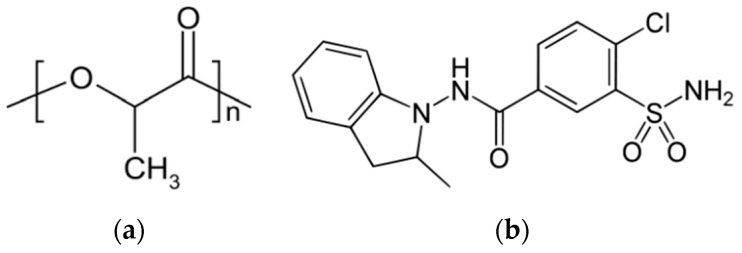
Chemical structure of (**a**) polylactide and (**b**) indapamide.

Polylactide (PLA; Figure 2a) is a non-active pharmaceutical ingredient: a biodegradable and biocompatible polymer containing 16.4% of the D-isomer, making it a fully amorphous material [24,25,26,52]. PLA was obtained from Cargill Dow Nature-Works LLC (Blair, NE, USA).

The indapamide (IND; Figure 2b; *M* = 365.835 g·mol^−1^) used in this study was obtained from Polpharma S. A. (Starogard Gdański, Poland). Indapamide was received as a white crystalline powder in the form of a hemihydrate of pharmaceutical grade. The degradation of the studied material begins around 260 °C [35], thus the highest temperature used in this study was 190 °C.

#### Sample Preparation

An amorphous sample of pure IND was prepared immediately prior to experimental measurements in DSC pan in the instrument, by heating the sample to 190 °C, holding it for 3 min, and then cooling it at a 10 °C·min^–1^ rate to room temperature. The PLA–IND system (mass ratio of 50/50) was prepared by a solvent evaporation method. The mixture was prepared several times to estimate the repeatability of the results. Amounts of 10–20 mg of IND and PLA were used in equal proportions and dissolved in 50 mL of chloroform, followed by evaporation of the solvent under reduced pressure at about 55–60 °C in a rotary evaporator. The polymer–API system was dried at 40 °C for two days. The obtained samples were in the form of a brittle, thin film.

### 2.2. Methods

Calorimetric measurements were performed using differential scanning calorimeters (DSC): DSC Q1000 and DSC 2920 from TA Instruments USA (New Castle, DE, USA). The temperature and heat-flow rate were calibrated with indium (*T*_m_ = 156.65 °C and Δ_f_*h* = 28.45 J·g^–1^) [41], and at least two tests were carried out on each sample. Sapphire (Al_2_O_3_) was used to calibrate the heat capacity [41].

Thin film samples of the PLA, PLA–IND system or powdered IND (2–10 mg) were placed into a standard aluminium pan with a pinhole. As a reference sample, an empty aluminium pan with a similar mass to that of the sample pan was used.

The standard DSC test included a heating run to 190 °C in order to erase thermal history, immediate cooling at a rate of 20 °C·min^–1^ to 5 °C followed by heating with a constant rate of 10 °C·min^–1^ to 190 °C.

#### 2.2.1. Determination of the Enthalpy Relaxation

To examine the isothermal physical ageing process, the samples, after heating to 190 °C, were cooled down (20 °C·min^–1^) to the chosen ageing temperature (*T*_a_) for ageing times between 10 min and 20 h. After ageing, the samples were immediately cooled to 5 °C at a 20 °C·min^–1^ rate, and the subsequent heating scan at a constant rate of 10 °C·min^–1^ provided the data for the aged sample.

Enthalpy relaxation was calculated from a heat-flow or heat capacity vs. temperature scan for aged and non-aged samples from the DSC measurement according to the Equation:

(1) $$\Delta h_{r}=\frac{1}{q}\overset{T_{2}}{\underset{T_{1}}{\int }}(\Phi_{\mathrm{aged}}-\Phi_{\mathrm{unaged}})dT=\overset{T_{2}}{\underset{T_{1}}{\int }}\left(c_{p\;\mathrm{aged}}-c_{p\;\mathrm{unaged}}\right)dT$$

where *q* is the heating rate, *T*_1_ and *T*_2_ are the limits of the integration temperature (where *T*_1_ < *T*_g_ < *T*_2_), *Φ*_aged_ and *Φ*_unaged_ are heat flows originating from the aged and unaged samples, respectively, and *c_p_* _aged,_ and *c_p_*_unaged_ are heat capacities of the aged and unaged samples, respectively.

Equilibrium enthalpy relaxation, $\Delta h_{r}^{\inf}$**,** values were calculated by using the Equation:

(2) $$\Delta h_{r}^{\inf}=\overset{T_{g}}{\underset{T_{a}}{\int }}\Delta c_{p}dT\approx \;\Delta c_{p}\;\left(T_{g}-T_{a}\right)$$

where Δ*c_p_* is the change of heat capacity in solid–liquid transition at a glass temperature and *T*_g_ and *T*_a_ are the glass transition and ageing temperature, respectively.

#### 2.2.2. Heat Capacity Calculations

The heat capacity (*c_p_*) of the PLA–IND (50/50) system was calculated according to the Equation:

(3) $$c_{p}=\frac{\Phi }{q}$$

where Φ is the measured heat-flow rate and *q* is the heating rate.

In order to compare the experimental heat capacity of the aged and unaged PLA–IND mixtures, the vibration heat capacity and the liquid heat capacity of examined samples were established. The heat capacity of the solid and liquid was estimated from a sum of linear combinations of the weight fractions of the solid and liquid heat capacity of PLA and IND [35,53]. The vibration of the heat capacity in the solid state of the PLA–IND (wt% 50/50) system, *c_p_*(vibration), was estimated by using equation [50]:

*c_p_*(vibration) = *x*^PLA^·*c_p_*^PLA^(vibration) + *x*^IND^·*c_p_*^IND^(vibration) (4)

where *x*^PLA^ and *x*^IND^ are the weight ratios, and *c_p_*^PLA^(vibration) and *c_p_*^IND^(vibration) are the vibrational heat capacities of PLA and IND in the solid state, respectively. The vibrational heat capacities of PLA and IND have already been published and were referenced in this study from [35,54].

The heat capacity of the liquid state for PLA–IND 50/50 (*w*/*w*) system was also calculated as a linear combination of two components as follows:

*c_p_*(liquid) = *x*^PLA^·*c_p_*^PLA^(liquid) + *x*^IND^·*c_p_*^IND^(liquid) (5)

where *x*^PLA^ and *x*^IND^ are the weight ratios, *c_p_*^PLA^(liquid) = 1.67 + 1.05 × 10^–3^ *T* [J·K^–1^·g^–1^] and was referenced from [54], while *c_p_*^IND^ (liquid) = 0.954 + 2.30 × 10^–3^ *T* [J·K^–1^·g^–1^] was referenced from [35].

#### 2.2.3. Determination of Fragility Parameter

The energy activation (*E*_a_) and fragility parameter (*m*) was obtained from the DSC scans using different cooling rates (0.2–10 °C·min^–1^) followed by heating with a constant heating rate of 10 °C·min^–1^. The measurements were performed within the temperature range of 25–190 °C. At 25 °C the sample was held for 5 min and at 190 °C for 3 min. The measurement from which the different cooling rates were used, the glass transition (during heating), is overlapped with the enthalpy relaxation peak caused by the physical ageing process. Due to this phenomenon, the glass transition temperature cannot be determined, so a fictive temperature is used to characterise a sample. In order to assess the fragility parameter, the value of *E*_a_ of the glass transition must be known. *E*_a_ was calculated based on the following Equation:

(6) $$-\frac{E_{a}\left(T_{g}\right)}{R}=\frac{d\left(\ln q\right)}{d\left(T^{-1}\right)}$$

where *q* is the heating/cooling rate, *R* is the gas constant and *T* is the fictive temperature (*T*_f_) in kelvin.

Knowing the value of activation energy, the fragility parameter can be described by the following Equation:

(7) $$m=\frac{E_{a}\left(T_{g}\right)}{2.303\cdot R\cdot T_{g}}$$

where *E*_a_ is the activation energy and *T*_g_ is the glass transition temperature in kelvin.

## 3. Results and Discussion

**Figure 3:**
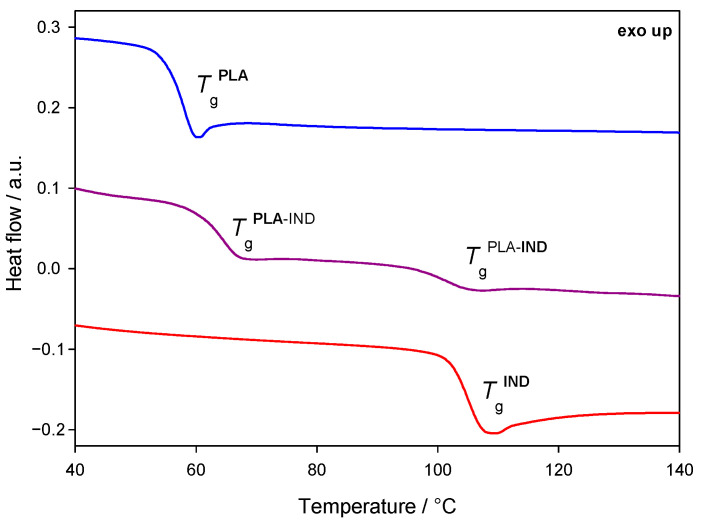
Heat-flow rate as a function of temperature for polylactide (PLA; blue curve), indapamide (IND; red curve) and the PLA–IND 50/50 (*w*/*w*) system (purple curve). Two glass transitions (*T*_g_) are observed for the PLA–IND system, indicating the immiscibility of the components.

Figure 3 shows the heat-flow rate as a function of temperature for PLA, IND and PLA–IND systems with a weight ratio of 50/50, obtained from the second heating scan with a heating rate of 10 °C·min^–1^. The glass transition of pure PLA is observed at 56.7 ± 0.8 °C. The amorphous form of IND was obtained by a rapid cooling of the sample from the molten state to a temperature below *T*_g_. The glass transition of the amorphous indapamide was observed at a temperature of 103.8 ± 1.1 °C and is in good agreement with the previous reports (102 °C) [35]. In the investigated PLA–IND 50/50 (*w*/*w*) system, two glass transitions were observed, the first at a temperature of 64.1 ± 0.3 °C, corresponding to the glass transition of PLA, and the second at 102.6 ± 1.1 °C, corresponding to the *T*_g_ of IND. The drug–polymer miscibility may be regarded as the “solubility” of the amorphous drug in the polymer. If the components are molecularly mixed (single amorphous phase), one glass transition is observed [55,56]. Two amorphous phases (two *T*_g_’s) were observed, indicating a lack of molecular miscibility between the components. Two-phase amorphous–amorphous solid dispersion is obtained during quenching. Similar behaviour has been observed for polylactide–valsartan and indomethacin–glucose systems [57,58]. Crystallisation and the melting of samples were not observed in IND and PLA–IND thermograms, suggesting the stability of the materials in the amorphous state against crystallisation even when heated up. The change of the heat capacity at the glass transition temperature for PLA was found to be 0.56 J·g^–1^·°C^–1^, for IND 0.44 J·g^–1^·°C^–1^, and for the PLA–IND (50/50) system 0.35 J·g^–1^·°C^–1^ and 0.145 J·g^–1^·°C^–1^ for the polymer and the drug, respectively (see Table 1). The changes in the heat capacity in the glass transition regions for the components in the PLA–IND system are lower than for the pure ingredients, disproportionate to mass ratios, which may indicate intermolecular interactions in the system. Otherwise, Δ*c*_p_ at *T*_g_ of the components in the system with a mass ratio of 50/50 would be two times lower than the Δ*c*_p_ in *T*_g_ for pure ingredients.

**Figure 4:**
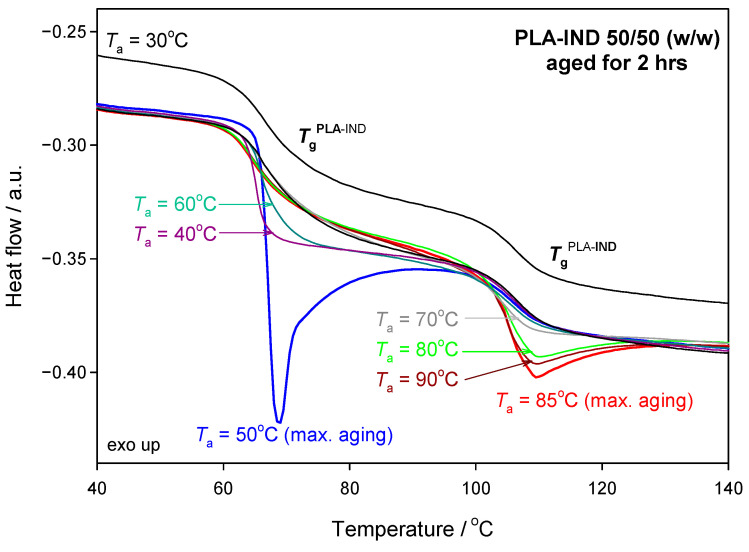
Heat-flow rate vs. temperature for the isothermal physical ageing showing enthalpy relaxation changes of the PLA–IND 50/50 (*w*/*w*) system after annealing for 2 h at different ageing temperatures, *T*_a_ = (30, 40, 50, 60, 70, 80, 85, 90) °C. The curve for the sample aged at 30 °C was shifted for clarity—no significant changes in enthalpy relaxation occurred.

Figure 4 shows a thermogram of PLA–IND 50/50 (*w*/*w*) for samples annealed for 2 h at different ageing temperatures below the *T*_g_ of IND, i.e., 30, 40, 50, 60, 70, 80, 85 and 90 °C. The annealing at 30 °C did not show any significant changes in enthalpy relaxation in the glass transition region of both the PLA and IND. The changes due to the physical ageing of IND in the PLA–IND system begin to be noticeable for ageing temperature (*T*_a_) at 70 °C and are the highest for *T*_a_ = 85 °C. For the annealing at 90 °C, enthalpy relaxation was lower than for 85 °C. This may be due to the fact that, at 90 °C, the material is already at the beginning of the glass transition region. The highest value of the enthalpy relaxation of IND for the PLA–IND 50/50 (*w*/*w*) system was observed for the physical ageing process at 85 °C and, therefore, the physical ageing process of the system for longer timescales was carried out at this temperature. It can also be observed that, in the PLA–IND 50/50 (*w*/*w*) system, PLA is aged the most at *T*_a_ = 50 °C while IND, after 2 h of annealing at this temperature, does not show any change. On the other hand, PLA at 70, 80, 85 and 90 °C is already in its liquid state and thus the physical ageing of the PLA polymer in PLA–IND 50/50 system at this temperature does not occur.

**Figure 5:**
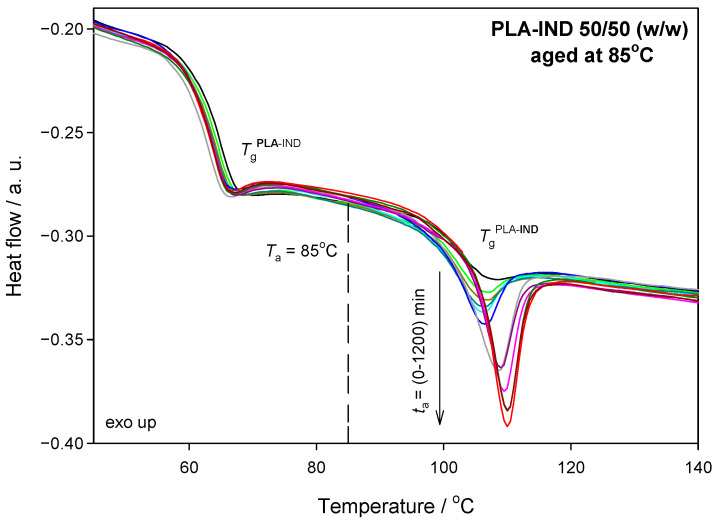
Heat-flow rate as a function of the temperature of the PLA–IND (50/50) system after annealing at 85 °C for various ageing times.

Figure 5 shows DSC curves for the PLA–IND system annealed at 85 °C for ageing times from 10 min to 20 h. It can be seen that the glass transition of polylactide remains unchanged as polylactide is already in a liquid state and, therefore, does not undergo physical ageing. IND underwent a physical ageing process and, as the ageing time increases, the enthalpy relaxation becomes larger and the maximum of the peaks also shifts towards higher temperature values.

**Figure 6:**
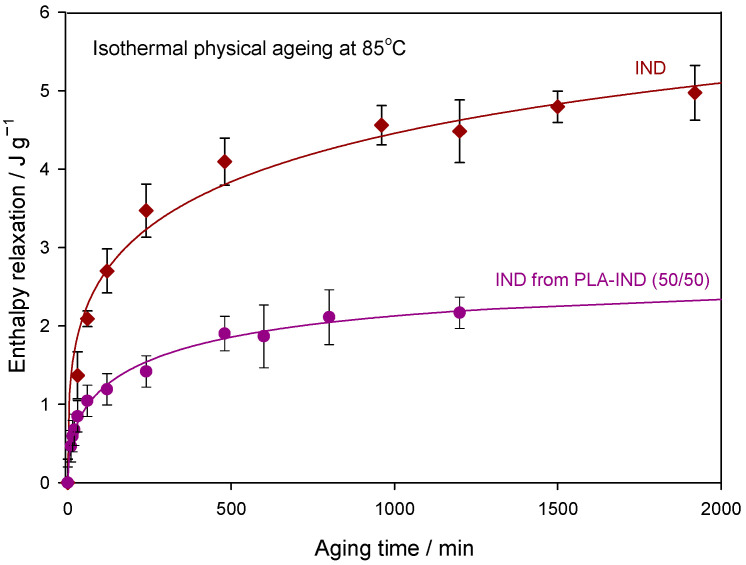
Experimental enthalpy relaxation for IND in the PLA–IND (50/50) system (dots) and for pure IND (squares) aged at 85 °C as a function of ageing time. The solid lines present the calculated enthalpy relaxation from the best fit of the experimental data to the KWW equation.

The enthalpy relaxation values of indapamide were calculated according to Equation (1), and the results were also compared with the results of the ageing of pure indapamide at the same ageing conditions as those in [36] (see Figure 6). The experimental enthalpy relaxations were fitted using the Kohlrausch–Williams–Watts (KWW) model [45,46], according to the relationship:

(8) $$\Delta h_{r}=\Delta h_{r}^{\inf}\cdot \left[1-\exp\left\{-{\left(t/\;\tau^{\mathrm{KWW}}\right)}^{\beta }\right\}\right]$$

where Δ*h*_r_ is measured enthalpy relaxation, $\Delta h_{r}^{\inf}$ is the equilibrium enthalpy relaxation, *t* is the ageing time, *τ*^KWW^ is the relaxation time, and *β* is the coefficient describing the distribution of relaxation times.

Figure 6 shows the experimental and calculated enthalpy relaxation (from the best fit) for pure IND and IND from the PLA–IND (50/50) system aged at 85 °C for various ageing times. Presented experimental results are the average values with error bars for the standard deviation (maximum of ±0.3 J·g^–1^) for pure IND (maximum of ±0.2 J·g^–1^) and for IND from the PLA–IND system. Initially, the changes in the enthalpy relaxation (*t*_a_) are noticeable (relatively significant), and, after about 4 h, the differences between relaxation enthalpies for subsequent ageing times decrease, and the value of enthalpy relaxation approaches the equilibrium enthalpy relaxation. The solid lines in Figure 6 represent the calculated enthalpy relaxation, Δ*h*_r_, from the best fit of experimental data to the KWW function (Equation (8)). From this best fit, the parameters of relaxation time *τ*^KWW^ and coefficient *β* were obtained. Both describe the kinetics of the physical ageing process of indapamide in a mixture of PLA and IND. The equilibrium enthalpy relaxation $\Delta h_{r}^{\inf}$ used in the KWW model (Equation (8)) was estimated according to Equation (2) using the data of Δ*c_p_*, *T*_a_ and *T*_g_ from Table 1. All of the calculated parameters from the KWW function are presented in Table 2.

Another parameter used for the description of the physical ageing kinetics is the recovery parameter, φ, which was obtained by transforming Equation (8) to the following form:

(9) $$\varphi \left(t\right)=\exp\left\{-{\left(\frac{t}{\;\tau^{\mathrm{KWW}}}\right)}^{\beta }\right\}=1-\;\Delta h_{r}/\Delta h_{r}^{\inf}$$

where all of the quantities in Equation (9) have the same meaning as in Equation (8).

The recovery parameter not only shows changes in enthalpy relaxation occurring during the isothermal physical ageing process but also shows how much more the system can change under given conditions and how fast the material reaches an equilibrium state. The recovery parameter can have values from 1 for non-aged material to 0 for material in which enthalpy relaxation corresponds to equilibrium enthalpy relaxation,$\;\Delta h_{r}^{\inf}$.

**Figure 7:**
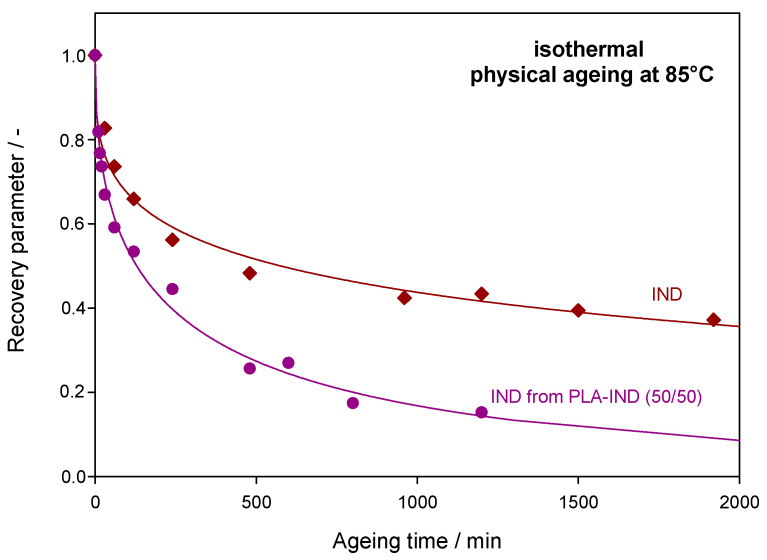
The recovery parameter, φ, values for IND in the PLA–IND (50/50) system (dots) and for pure IND (squares) aged at 85 °C as a function of ageing time. Solid lines represent the fit of the calculated data to Equation (9).

Figure 7 shows the recovery parameter versus the ageing time (*t*_a_) for the results of enthalpy recovery presented in Figure 6. The solid lines in Figure 7 present the calculated recovery parameter according to Equation (9) with parameters of *τ*^KWW^ and *β* obtained from the best fit of the experimental data of Δ*h*_r_ to the KWW Equation (8).

According to the values of *τ*^KWW^ (see Table 2), the recovery parameter lowered much faster for IND in the PLA–IND system than for pure IND. As can be seen, IND in the mixture achieves equilibrium faster than pure API despite lower relaxation enthalpy values. Thus, the changes in enthalpy caused by physical ageing are lower and go down faster for the drug in the system with polylactide compared with the pure API. For IND from the mixture, Δ*h*_r_ and φ reach a saturation relatively quickly and, after 1200 min (20 h), further changes in the enthalpy relaxation are not as significant. Additionally, the recovery parameter after 1200 min of annealing at 85 °C reaches a value of around 0.1, which is close to the equilibrium value. The enthalpy relaxation for pure IND after 1200 min reaches 50% of the equilibrium enthalpy relaxation. It can therefore be assumed that adding the PLA to amorphous IND results in an improvement of the physical stability (in terms of the physical ageing) of the drug because, after 2400 min (40 h) at ageing temperature, further changes of the enthalpy relaxation are very small and therefore changes in physicochemical properties are unlikely to occur. The relaxation time of IND aged at 85 °C is nearly six times higher for the pure drug than for the drug in the PLA–IND (50/50) system. The *β* parameter may provide a measure of the physical stability of amorphous systems [59]. The glass formers with a broader distribution of structural relaxation times (low *β* value) may be more susceptible to nucleation resulting in reduced physical stability [60].

The non-isothermal physical ageing of PLA–IND (wt% 50/50) was characterised by activation energy (*E*_a_) and fragility parameter (*m*).

**Figure 8:**
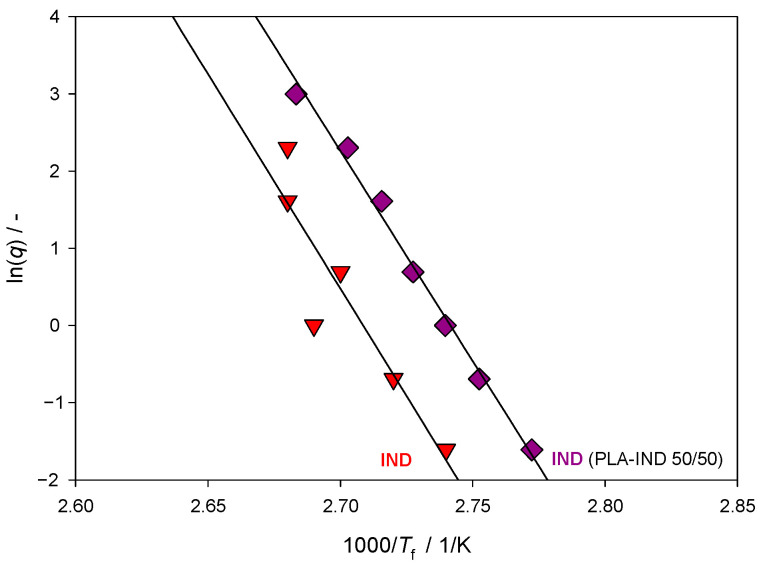
Arrhenius plots of temperature dependence on the cooling rates of the IND in the PLA–IND (50/50) system and of pure IND.

Figure 8 presents the dependence of the fictive temperature derived by reheating the sample after the experiment with varying rates of cooling together with the linear regression line. The slopes in Figure 8 give the activation energy, *E*_a_, according to Equation (6). Good linearity is observed between the temperatures and cooling rate. The slopes of the Arrhenius plots representing ln(*q*) as a function of 1/*T* were calculated using the least-square linear regression. The activation energy (*E*_a_) and the value of kinetic fragility (*m*) were determined based on Equations (6) and (7), respectively (Table 3).

Relatively low values of the fragility parameter indicate that IND and its system with PLA belong to materials classified as ‘moderately fragile’. A value of *m* ≈ 200 denotes considerable fragile behaviour, while a value of *m* ≈ 16 and under indicates a strong glass. Most compounds of pharmaceutical interest have a value of fragility parameter between these two extremes, that is, 16 < *m* < 200, and are categorised as “moderately fragile” [47].

**Figure 9:**
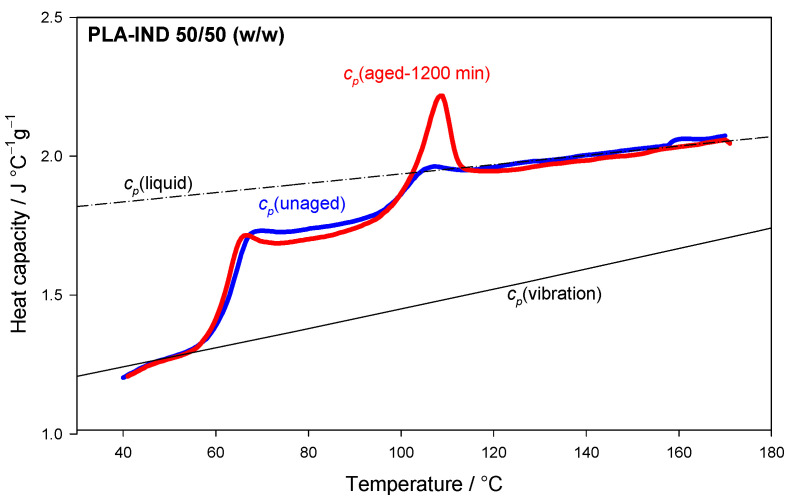
Heat capacity of the PLA–IND (50/50) system studied by DSC, where *c*_p_(aged-1200 min) and *c*_p_(unaged) represent the experimental heat capacity of the aged and unaged sample, *c_p_*(vibration) represents the vibrational heat capacity of the PLA–IND (50/50) system and *c_p_*(liquid) represents the heat capacity of the liquid state of PLA–IND (50/50).

Figure 9 presents the heat capacity as the function of the temperature of the unaged and aged PLA–IND (50/50) system for 1200 min at 85 °C. The experimental data of *c_p_*(unaged) and *c_p_*(aged-1200 min) for heat capacity were compared with the reference *c_p_* for vibrational, solid heat capacity—*c_p_*(vibration)—and liquid heat capacity—*c_p_*(liquid)— of the PLA–IND 50/50 system. The heat capacity of the solid and liquid state of the tested mixture was calculated using Equations (4) and (5). Good agreement between the experimental data and the calculated heat capacity of the PLA–IND system was observed below and above glass transition regions.

## 4. Conclusions

The isothermal and non-isothermal physical ageing processes of IND in a PLA–IND system with a 50/50 mass ratio and pure IND were studied using differential scanning calorimetry. The study demonstrated that the IND and the PLA in the PLA–IND system are immiscible for a mass ratio of 50/50, exhibiting two distinct *T*_g_’s. The presence of PLA in a 50/50 system impacts the kinetics of the physical ageing process of the API.

The isothermal physical ageing process of IND in the PLA–IND system was studied at various ageing temperatures (30–90 °C) for an ageing time of 2 h. It was shown that the physical ageing process of IND in the system at *T*_a_ = 85 °C is characterised by the highest enthalpy relaxation. Moreover, the results were compared with data for the physical ageing process at 85 °C for pure IND. The values of the enthalpy relaxation of IND from the PLA–IND (50/50) system were lower than those for pure IND. The experimental data of enthalpy relaxation were fitted to the KWW equation. Relaxation time for IND from the mixture is lower than that for pure IND, which may indicate a decrease in the stability of the drug. Furthermore, IND in the system with PLA, compared with pure IND, reaches a saturation of enthalpy relaxation relatively quickly and, after 20 h at 85 °C, further changes in the enthalpy relaxation due to physical ageing are not noticeable, while the recovery parameter has a value of around 0.1, which is close to the equilibrium.

Non-isothermal physical ageing experiments of IND and IND from the PLA–IND (50/50) system were also carried out. The results show similar values of the activation energy and fragility parameter for IND from the system with PLA compared with the pure IND.

The heat capacity of the PLA–IND system was determined based on the contribution of heat capacities of the components: polylactide and indapamide. The heat capacity of the solid and liquid was estimated from a sum of the linear combinations of the mass fractions of the solid and liquid heat capacity of PLA and IND, respectively. A good agreement between the experimental data and the calculated heat capacity of the PLA–IND system was observed.

## Figures and Tables

**Table 1:** The glass transition temperature (*T*_g_) and changes of the heat capacity (Δ*c_p_*) at *T*_g_ for PLA, IND and the PLA–IND 50/50 (*w*/*w*) system.

Sample (w%/w%)	*T*_g_ ± SD/°C	Δ*c_p_* at *T*_g_ ± SD/J·g^–1^·°C^–1^
PLA (100/0)	56.7 ± 0.8	0.56 ± 0.01
PLA–IND (50/50)	64.1 ± 0.3/102.6 ± 1.1	0.35 ± 0.01/0.145 ± 0.01
IND (0/100)	103.9 ± 1.1	0.44 ± 0.01

**Table 2:** KWW parameters for pure IND and IND from the PLA–IND 50/50 (*w*/*w*) system aged at 85 °C.

Sample	$\Delta h_{r}^{inf}\;$/J·g^−1^	β/–	$\tau^{KWW}\;$/h
IND *	7.92	0.33	28
IND in the PLA–IND (50/50) system	2.56	0.46	4.75

* taken from Ref. [36].

**Table 3:** Energy activation and fragility parameters of the IND from the PLA–IND (50/50) system and of pure IND obtained from the dependence of *T*_f_ on heating rate.

Sample	Energy Activation, Δ*E*_a_ ± SD/kJ/mol	Fragility Parameter, *m* ± SD/–
IND in the PLA–IND (50/50) system	452.5 ± 18.5	63 ± 3
IND	473 ± 51	66 ± 7

## Data Availability

Available on request from authors.
